# Supplementary material for: Patterns of Genetic Diversity among Alphasatellites Infecting Gossypium Species
Source: Pathogens. 2022 Jul 4;11(7):763. doi: 10.3390/pathogens11070763 (PMC9319557; doi:10.3390/pathogens11070763)
Supplement: Supplementary file 1 [file pathogens-11-00763-s001.zip › Supplementary files/Supplementary TableS1.pdf]

Table S1: Average *P*-values of recombination events for different alphasatellites. Recombinant regions (a to i) are shown on supplementary figure-2. RDP4 was used to identify the average P-values of possible parental regions. A cut off *P*-value 0.05 was used throughout the analysis.

**RDP Modules**	**Event**	**Break point**	**Average P values**
Maxchi<br>SciScan<br>GENECONV<br>Bootscan<br>Chimaera<br>RDP	a	1-106	4.91×10^{-2}<br>$9.471 \text{ X } 10^{-21}$<br>$1.40 \text{ X } 10^{-3}$<br>$1.106 \times 10^{-4}$<br>-<br>-
Maxchi<br>SciScan<br>GENECONV<br>Bootscan<br>Chimaera<br>RDP	b	470-750	1.346×10^{-10}<br>1.808×10^{-17}<br>1.329×10^{-15}<br>4.506×10^{-22}<br>2.379×10^{-5}<br>1.719×10^{-20}
Maxchi<br>SciScan<br>GENECONV<br>Bootscan<br>Chimaera<br>RDP	c	50-188	1.08×10^{-5}<br>2.210×10^{-15}<br>1.978×10^{-22}<br>1.936×10^{-18}<br>2.441×10^{-3}<br>3.034×10^{-15}
Maxchi<br>SciScan<br>GENECONV<br>Bootscan<br>Chimaera<br>RDP	d	189-1067	3.284×10^{-5}<br>-<br>-<br>-<br>2.069×10^{-4}<br>1.719×10^{-2}
Maxchi<br>SciScan<br>GENECONV<br>Bootscan<br>Chimaera<br>RDP	e	1069-66*	1.635×10^{-03}<br>3.344×10^{-10}<br>-<br>2.055×10^{-03}<br>3.344×10^{-10}<br>3.44×10^{-2}
Maxchi<br>SciScan<br>GENECONV<br>Bootscan<br>Chimaera<br>RDP	f		-
Maxchi<br>SciScan<br>GENECONV<br>Bootscan<br>Chimaera<br>RDP	g	176-430	5.035×10^{-15}<br>2.026×10^{-20}<br>4.199×10^{-18}<br>5.068×10^{-16}<br>6.869×10^{-9}<br>3.499×10^{-03}
Maxchi<br>SciScan<br>GENECONV<br>Bootscan<br>Chimaera<br>RDP	h	430-726	1.938×10^{-16}<br>1.216×10^{-13}<br>8.749×10^{-14}<br>2.640×10^{-16}<br>1.136×10^{-3}<br>1.328×10^{-4}
Maxchi<br>SciScan<br>GENECONV<br>Bootscan<br>Chimera<br>RDP	i	490-1066	2.687×10^{-6}<br>$4.448 \times 10\text{-}^{14}$<br>-<br>$4.178 \times 10^{-5}$<br>$6.068 \times 10^{-8}$<br>$2.828 \times 10^{-5}$

Footnotes for table-1. * Includes the origin of replication.
