# Supplementary figures and images for: Patterns of Genetic Diversity among Alphasatellites Infecting Gossypium Species
Source: Pathogens. 2022 Jul 4;11(7):763. doi: 10.3390/pathogens11070763 (PMC9319557; doi:10.3390/pathogens11070763)

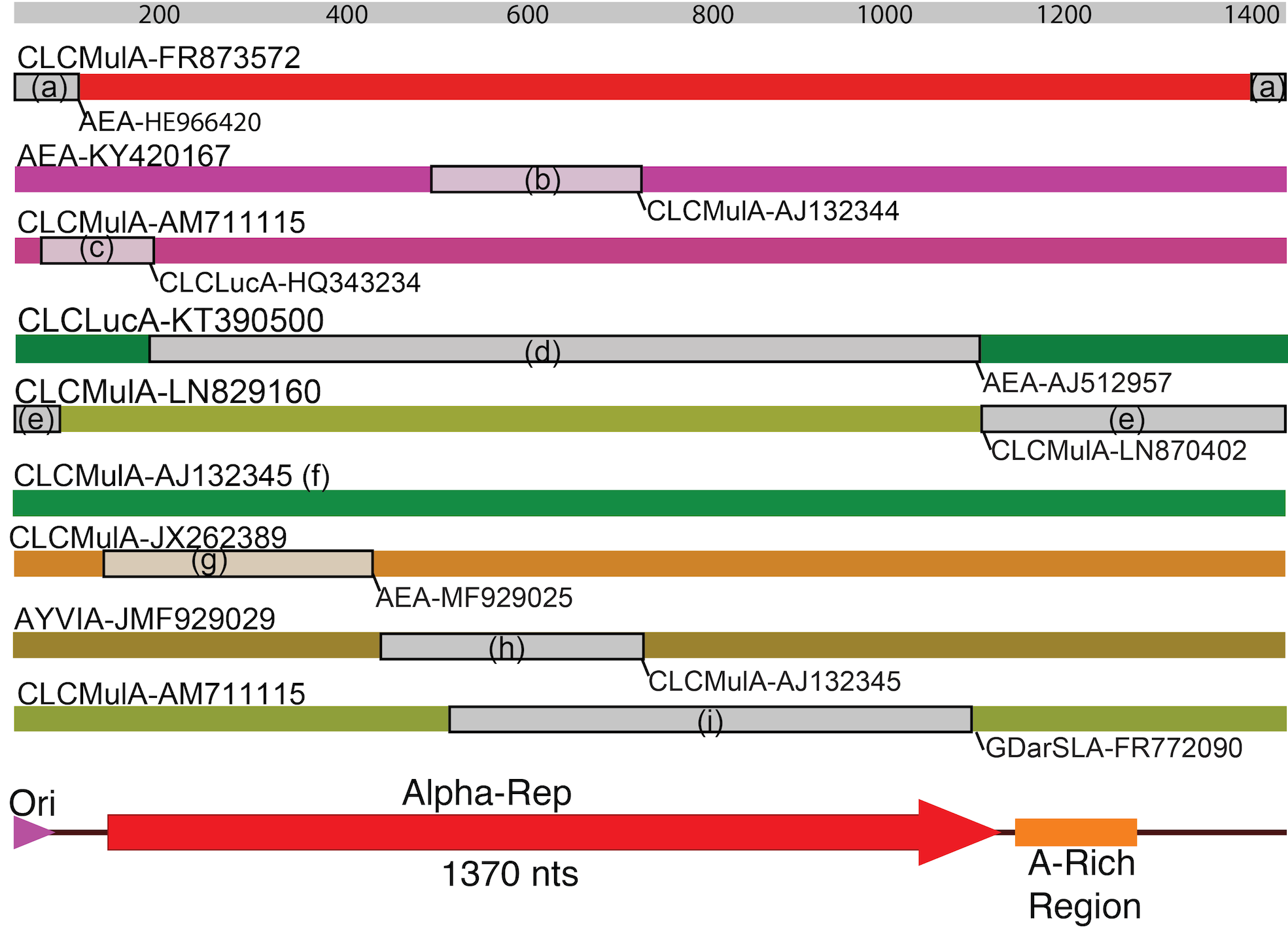

Supplement: Supplementary file 1 [file pathogens-11-00763-s001.zip › Supplementary files/Supplementary Figure S2.png]
